# Therapeutic potential of combating cancer by restoring wild-type p53 through mRNA nanodelivery

**DOI:** 10.1016/j.nano.2024.102732

**Published:** 2024-01-08

**Authors:** Divya Kamath, Tomoo Iwakuma, Stefan H. Bossmann

**Affiliations:** a The University of Kansas Medical Center, Department of Cancer Biology, 3901 Rainbow Blvd, mailstop 1071, 66160 Kansas City, KS, USA; b Children’s Mercy Hospital, Adele Hall Campus, 2401 Gillham Rd, Kansas City, MO 64108, USA

**Keywords:** p53 mutant, cancer, Gene therapy, Nanotechnology, Restoring wild-type p53

## Abstract

Among the tumor suppressor genes, *TP53* is the most frequently mutated in human cancers, and most mutations are missense mutations causing production of mutant p53 (mutp53) proteins. *TP53* mutations not only results in loss of function (LOH) as a transcription factor and a tumor suppressor, but also gain wild-type p53 (WTp53)-independent oncogenic functions that enhance cancer metastasis and progression (Yamamoto and Iwakuma, 2018; Zhang et al., 2022). *TP53* has extensively been studied as a therapeutic target as well as for drug development and therapies, however with limited success. Achieving targeted therapies for restoration of WTp53 function and depletion or repair of mutant p53 (mutp53) will have far reaching implication in cancer treatment and therapies. This review briefly discusses the role of p53 mutation in cancer and the therapeutic potential of restoring WTp53 through the advances in mRNA nanomedicine.

## Introduction

Cancer is a disease characterized by accumulated genetic alterations, which are caused by exposure to stressors (internal & external) and/or sporadic genetic changes, promoting malignant progression.^3^ Each tumor can be characterized by distinctive permutation of alterations both genetic and epigenetic. Genome-wide studies support that all tumors are heterogenous, and not all alterations have the same effect.^4^ Genes that regulate pathways for cell survival, cell fate, and genome maintenance are classified as driver genes, whereas genes that do not affect significant cellular pathways are called as passenger genes. Driver genes harboring nonsynonymous mutations elevate the risk of tumorigenesis, compared to passenger genes.^5,6^ Oncogenes, tumor suppressor genes, and DNA repair genes are classified as cancer drivers.^4^ The tumor suppressor genes produce proteins that regulate cell division, proliferation, genome integration, and cell death. They are considered as gate-keepers against cancer and often require mutations in both alleles to increase the rate of cancer occurrence.^7^

Mutations in *TP53* occur in more than 50 % of cancer. P53 protein, encoded by the *TP53* gene, acts as a nuclear transcription factor, and lead to a cascade of genetic alternations and malignant progression.^8,9^ WTp53 protein binds to consensus sequences in the genome to regulate numerous downstream target genes mainly involved in cell cycle, cell proliferation, cell death, and cell metabolism. WTp53 is also considered as the guardian of the genome as it plays a critical role in the DNA damage response and repair, in addition to preventing tumorigenesis.^10^ Homeostatic cells express WTp53 in a low concentration. Upon cellular stress WTp53 is post-translationally modified leading to its stabilization and activation in the cells. Nuclear WTp53 binds to the DNA consensus sequences of different downstream target genes, likely based on the stressors, to regulate their gene expression.^11^ Thus, WTp53 can prevent proliferation of cells with damaged genome by either inducing cell cycle arrest to repair damaged DNA or by eliminating the cells by apoptosis.^10,12^ Such WTp53 function offsets the wide range of genomic damages, thereby preventing accumulation of mutations that trigger tumorigenesis.^13^

## WTp53 protein encompasses three critical domains

**Fig. 1.**
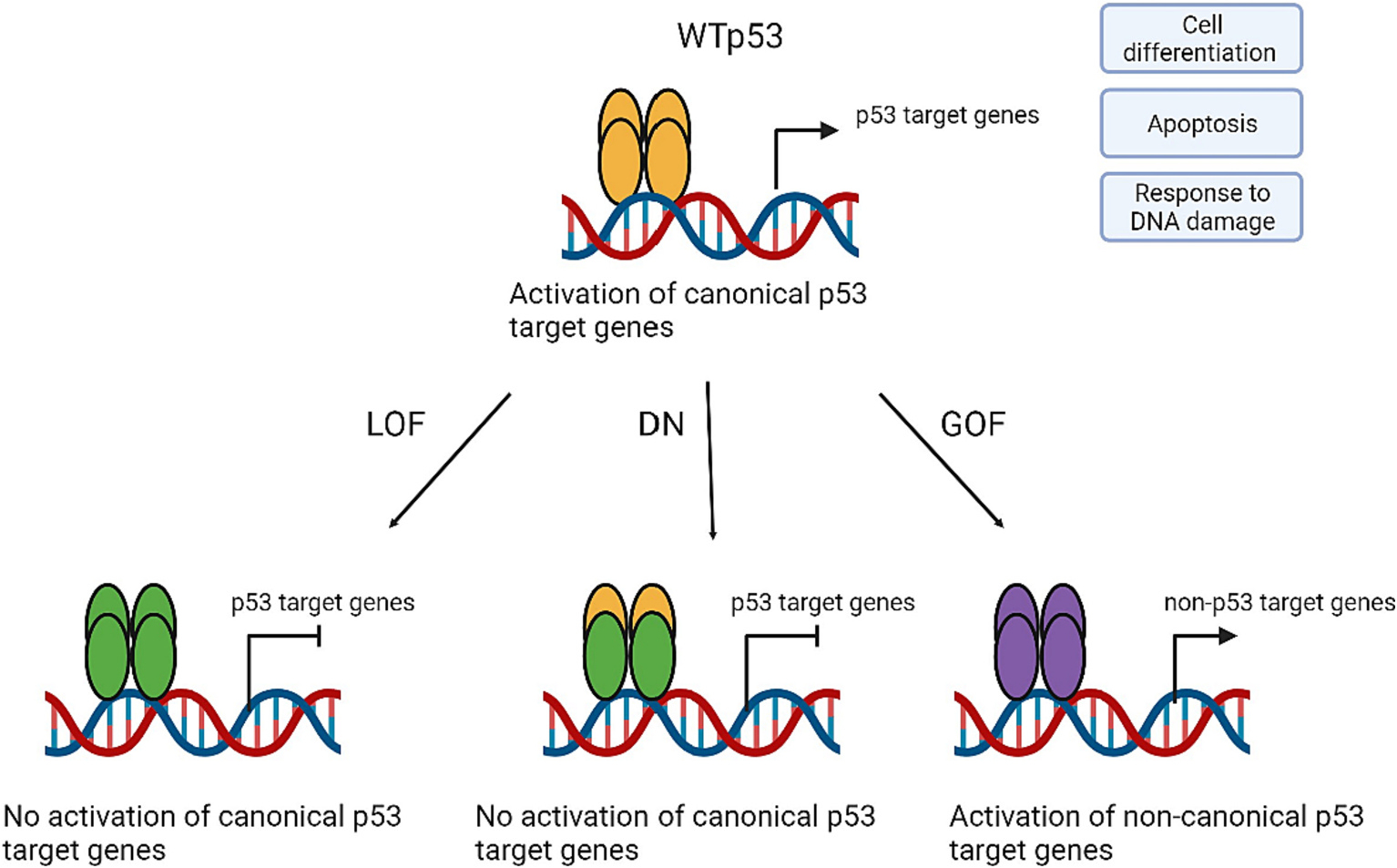
Created with BioRender.com Role of mutp53 in cancer: WTp53 activates a variety of genes responsible for cell differentiation, apoptosis, and DNA damage control. These gene products prevent accumulation of abnormal cells. Alternations in *TP53* can result in 3 types of mutations each with different characteristics. The loss of function (LOF) and the dominant negative mutation (DN) prevents activation of canonical WTp53 target genes whereas the gain of function (GOF) mutations result in activation of non-canonical p53 target genes resulting in cellular imbalance.^22^

Structurally, the WTp53 protein encompasses three critical domains; N-terminal transactivation domain, DNA binding domain, and C-terminal oligomerization domain. Approximately 95 % of mutations in *TP53* occur in the DNA binding domain, of which the majority are missense mutations causing amino acids changes.^13,14^ These changes either interfere with the WTp53’s DNA-binding activity or modify p53’s structure leading to disruption of the WTp53’s DNA-binding ability.^15^ Thus, missense mutp53 is defective in binding to the consensus DNA sequences required for transcriptional activation of WTp53 target genes, thereby showing loss of function (LOF) phenotypes (Fig. 1).^9,14,16^ Since missense mutp53, which is translated as a full-length protein defective in the DNA-binding ability, fails to transactivate its own E3 ubiquitin ligase, MDM2, mutp53 often accumulates in the nucleus of the tumor cells.^17,18^ Consequently, the accumulated mutp53 forms a hetero tetramer with existing WTp53 to inhibit WTp53 function, thus having the dominant negative (DN) activity. Moreover, missense mutp53 not only loses the function of WTp53, but also acquires new abilities to promote malignant progression independent of WTp53. Such oncogenic function of mutp53 is referred to as gain of function (GOF) which increases the tumor malignancy, metastasis, and drug resistance.^1,8,19,20^ Hence, the presence of missense mutp53 in tumors is well-correlated with adverse clinical outcomes and poor prognosis in patients.^2,9,21^ The LOF, DN, and GOF activities of missense mutp53 all contribute to promote immortalization and transformation of normal cells to accelerate malignant progression.^11,13^

### Mutated p53 is an excellent target in cancer therapy

**Fig. 2.**
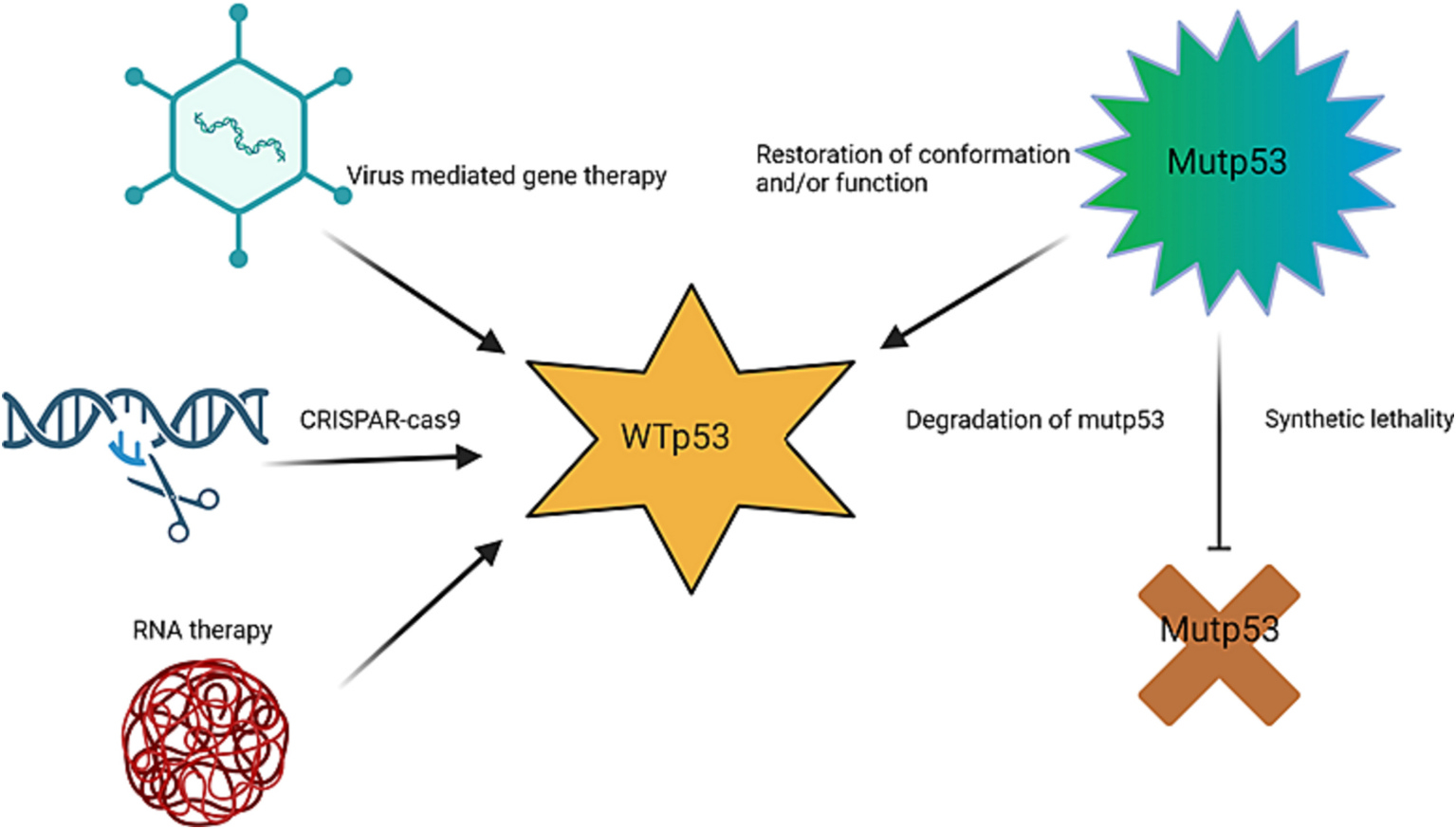
Created with BioRender.com Strategies to target p53 mutations: Mutp53 can lead to imbalance in the cells resulting in malignant transformation of the cell. The strategies to restore the activity of mutp53 include restoring conformation or function of mutp53, degradation of mutp53, and synthetic lethality. There are also efforts of exogeneous restoration of the *TP53 gene* or RNA to restore the function of WTp53. This includes use of viral vectors, the CRISPAR-cas9 technology, and/or mRNA therapy.

Since mutp53 is frequently found in tumors and rarely found in normal cells, mutp53 appears to be an ideal target for cancer therapy. However, initially, p53 was not considered an easy druggable target, on account of it being a nuclear transcription factor.^23^ Three decades of research later a lot of advancements have been made towards p53-based therapies. Current therapies focus on mainly three strategies; (i) restore conformation and function of existing mutp53 to those of WTp53, (ii) deplete missense mutp53, and (iii) induce p53 synthetic lethality (Fig. 2).^9,13^ A recent review by Hu et al.,^24^ “Targeting mutant p53 for cancer therapy: direct and indirect strategies” gives a comprehensive overview of the strategies developed and currently used. Despite all the advances, there are currently no FDA-approved therapies targeting either form of p53 yet.^2,13,25,26^

Evidence for mouse models shows that restoration of endogenous WTp53 activity can lead to regression of tumors. Hence, there has been more focused efforts on restoring WTp53 expression and function in cancer cells using therapeutic nucleic acids (NA) like DNA, siRNA, mRNA, and miRNA (Fig. 2).^15,27–30^ Gene therapy using replication incompetent adenovirus as a delivery vehicle encoding WTp53 cDNA has been previously tested with limited success.^31^ Bossi et al. (2007) in their review discuss details of gene therapy studies for WTp53 expression.^31^ Currently, Gendicine (rAD-p53), a recombinant adenoviral vector encoding human WTp53, is the only gene therapy treatment that has been approved in China for head and neck cancer,^32–34^ but its use is limited. The major drawbacks in these methods include expense, toxicity, immunogenicity, risk of random genome integration, and off target effects. In addition, delivery of these reagents to all tumor cells is the major hurdle of the gene therapy field. Recently, the CRISPR/Cas9 gene editing technique has been gaining momentum for replacement for mutp53 sequences with WTp53 sequences, providing more stable gene replacement or repair.^2,13,35^ However, the risk of random gene integration and off target effects, in addition to gene delivery-associated issues, remain.

## Recent advances in mRNA-based therapy

Recent advances in mRNA-based therapy alleviate some of the concerns encountered with genetic therapy or drug-based protein correction/restoration therapies. RNA therapy basically involves delivery of mRNA, produced in a cell free system *in vitro,* into the cytoplasm of the target cells. Since mRNA is translated to a protein in the cytoplasm of the cells, the delivered mRNA does not need to enter the nucleus, which could eliminate the risk of genome integration. Although it expresses the protein of interest like therapeutic DNA,^30,36^ mRNA is more susceptible to degradation by nucleases and is also considered to be more immunogenic than DNA. These sensitivities of mRNA can be minimized by specifically designing and tailoring the sequences *in vitro*.

**Fig. 3.**
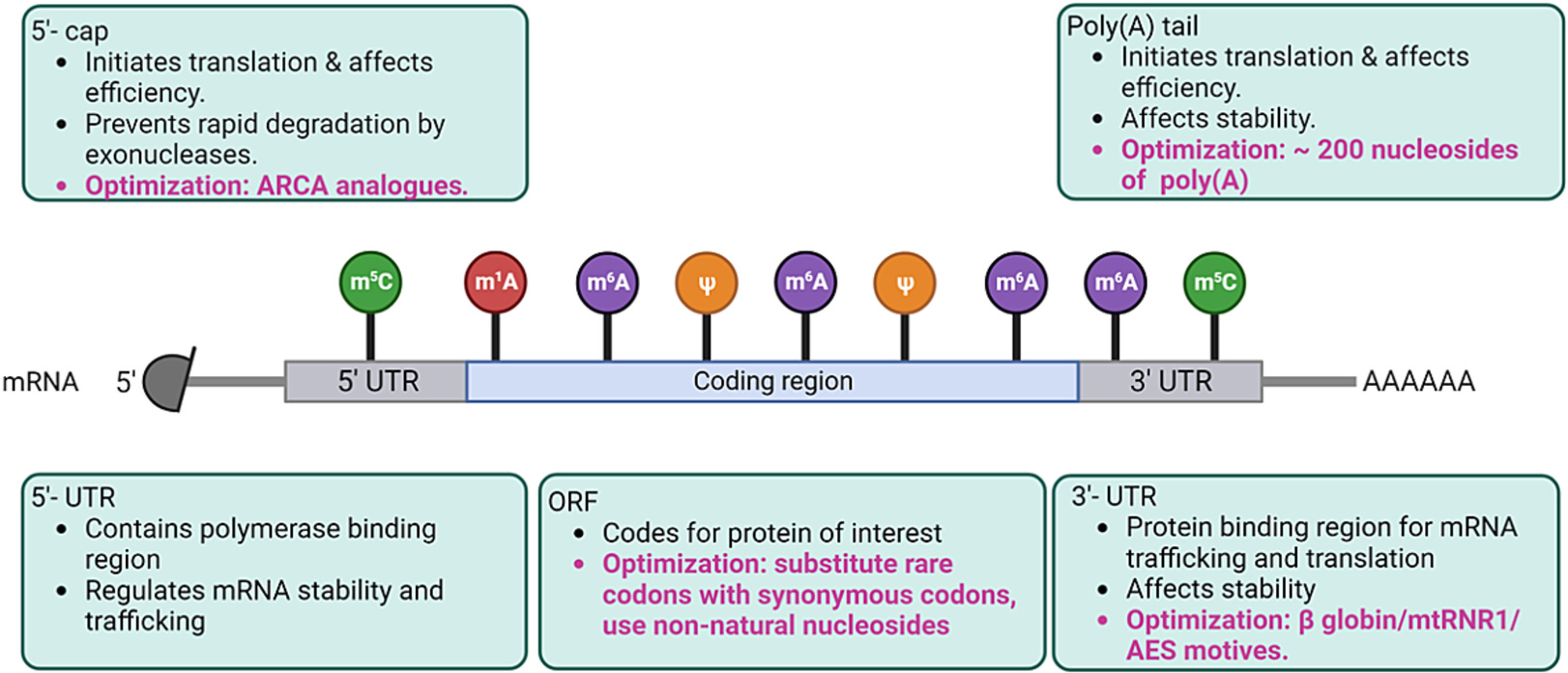
Created with BioRender.com mRNA structure and optimization. mRNA is a negatively charged molecule with distinctive regions. Each distinctive region serves specific purpose which can be optimized for mRNA to be used therapeutically.

mRNA is single stranded tripartite molecule consisting of the 5′ untranslated region (UTR), the coding region and the 3′ untranslated region (UTR) (Fig. 3). The 5′UTR consists of critical regions dictating translation initiation, translation efficiency, and protecting the mRNA from degradation by the 5′-3′ nucleases. It encompasses the Kozak’s sequence and the polymerase binding region.^37^ It also holds the 5′cap structure. Chemically, the core structure of the 5′cap (cap0) is an inverted 7-methylguanosine linked *via* a 5′-5′ triphosphate bridge to the first transcribed nucleotide. Based on the degree and location of methylation, the cap structure varies to form cap1 and cap2.^38^ Recent advances in the invention of trinucleotide cap analog (eg. ARCA – Anti-Reverse Cap Analog) have increased translational efficiency even further as discussed in the review by Kore et al.^39^ Based on the cell specification the cap structure *in vitro* can be customized to maximize translation efficiency. The 3′UTR, analogous to the 5′UTR, influences mRNA stability and translational efficiency. The 3′UTR region in eukaryotic transcripts contains poly(A) tail consisting of an average of 200 nucleotide of adenosine monophosphates. The poly(A) tail recruits the poly (A) tail binding proteins (PABPC) together with the other initiation factors, and the mRNA cap forms a closed loop structure for initiation of translation. The PABPC are also considered to protect the 3′end of the mRNA from exonucleases, thereby contributing to the stability of the mRNA.^37,40^ The length of the poly(A) tail can be customized *in vitro* to ~200 nt to enhance translation efficiency. The 3′UTR region consists of A + U rich elements (ARE) that speed up the mRNA decay process.^41^ The synthetically produced mRNAs can be redesigned to reduce ARE sequences to improve the stability and add sequences like the mtRNR1 (mitochondrially encoded non-coding 12S rRNA) and AES (amino terminal enhancer of split) sequences to increase the translation efficiency.^42^

The UTR regions affect the mRNA stability, translation initiation and efficiency. The primary mRNA coding sequence affects the half-life and the rate of elongation of translated peptide (Fig. 3). The codon region may contain non-optimal codons that can lead to stalling of the ribosomes, improper protein folding, and/or lower protein yield. This can be overcome by optimizing the coding sequences. The genetic code is degenerate, and many amino acids are encoded by synonymous codons (*i.e.*, different codons code for same amino acids). This can be used to design the DNA template containing optimal codons positively correlating with abundant tRNAs and eliminating rare codons that can stall or reduce rate of translation. Different programs are currently available to optimize the codon for faster rate and higher protein yields.^43,44^ Thus, mRNA can be relatively easily tailor-made, chemically modified, and synthesized, making mRNA a superior molecule to be used as therapeutics.^45–47^

Despite these advantages, mRNA cannot pass by itself through the negatively charged membrane due to repulsive forces. Also, once inside the cell the naked mRNA can be degraded by the enodnucleases and may also elicit immunogenic response. For these reasons the delivery vehicle to carry mRNA inside the cells is extremely critical and needs to be well designed.^48^ Ideally the delivery vehicle should deposit the RNA in the cytoplasm protecting it during entry and transport. It should also compensate for the inherent hydrophobicity and negative charge of the mRNA.^45^ For these reasons, non-viral, nano scaled carriers consisting of either lipid, polymer or lipid-polymer hybrid have gained a lot of traction.^49^ Most nano-carriers can be made to target specific cells or are eliminated after a while by the cellular machinery. These characteristics of the nanocarrier thereby mitigate systemic toxicity and build-up of the carrier molecule reducing overall toxicities associated with such therapies. Recent reviews by Cabral et al. (2020)^50^ and Fuente et al. (2022)^48^ discuss the most current advances in mRNA nanomedicine.

Recently published research corroborates the potential of nanocarrier-delivered *WTp53*-mRNA in non-small cell lung cancers (NSCLC) and in hepatocellular carcinoma (HCC). Nanoparticle (NP)-delivered *WTp53*-mRNA was able to slow the growth of these cells by inducing cell cycle arrest and apoptosis.^51^ This is a crucial finding as high WTp53 expression has been correlated with improved patient survival in both HCC and NSCLS patients as per the clinical data in TCGA.^52,53^ Moreover, Shi et al.^54^ evaluated and optimized a CXCR4-targeted *WTp53*-mRNA system to induce p53 expression in HCC. Using p53-null murine models they show that *WTp53*-mRNA can change the tumor microenvironment. An anti-PD1 therapy in combination with *WTp53*-mRNA-NP showed changes in the immune microenvironment enhancing recruitment of activated effector T cells.^55^

Overall, these pioneering studies show the potential of using NP-delivered *WTp53* mRNA as a therapeutic strategy alone or in combination. However, when the DN and GOF activities of mutp53 are considered, supplementing the cells with WTp53 alone may not be enough to efficiently induce WTp53-mediated tumor suppression. It would be necessary to deplete mutp53 to maximize the introduced or restored WTp53 activity. This can be done using existing drugs like statins or using mutp53-specific small RNA like siRNAs.^56,57^ When WTp53 is introduced in cells depleted for mutp53, the WTp53 activity may be efficiently increased to the threshold level necessary to inhibit tumor cell proliferation, which would increase the chances of delaying malignant progression and/or preventing cancer cells from acquiring drug resistance.

Because of the ease of modifying mRNA to overcome its limiting factors, the potential of mRNA therapeutics in cancer and in other diseases is endless. Song et al. (2022) and Wu et al. (2023) provide a comprehensive list of currently developing and upcoming clinical trials for mRNA-based therapeutics in different diseases.^58,59^ As can be seen in these reviews the potential of mRNA nanomedicine is not restricted to its therapeutic potential alone. The success of *SARS-CoV* mRNA vaccine highlights its prophylactic potential and has been emulated in other diseases as well. In cancers too, NPs carrying tumor suppressors and mRNA vaccines would help inhibit or delay cancer progression. Since oncogenes are present in multiple types of cancer, restoring the function of the guardian of the genome and meanwhile depleting oncogenes may be an efficient strategy to improve current anti-cancer therapies as well as survival for patients with cancers. Reviews by Kranz et al.^60^ and Breckpot et al.^61^ discuss the clinical landscape of mRNA-based cancer immunotherapy.

## Conclusion

The potential of mRNA nanomedicine is boundless. The ability to customize mRNA unlocks new avenues to personalized medicine in combination with advances in genetic testing. It holds immense potential to not only change the landscape of cancer and other diseases but also brings hope to people facing limited treatment options.
